# Soybean locus qDTF-7 as an example of genetic heterogeneity associated with flowering and maturity time

**DOI:** 10.18699/vjgb-26-06

**Published:** 2026-03

**Authors:** R.N. Perfil’ev, M.I. Shmatova, A.B. Shcherban, E.A. Salina

**Affiliations:** Institute of Cytology and Genetics of the Siberian Branch of the Russian Academy of Sciences, Novosibirsk, Russia; Institute of Cytology and Genetics of the Siberian Branch of the Russian Academy of Sciences, Novosibirsk, Russia; Institute of Cytology and Genetics of the Siberian Branch of the Russian Academy of Sciences, Novosibirsk, Russia Kurchatov Genomic Center of ICG SB RAS, Novosibirsk, Russia; Institute of Cytology and Genetics of the Siberian Branch of the Russian Academy of Sciences, Novosibirsk, Russia Kurchatov Genomic Center of ICG SB RAS, Novosibirsk, Russia

**Keywords:** soybean, flowering, TOE1, RVE8, genetic heterogeneity, cоя, цветение, TOE1, RVE8, генетическая гетерогенность

## Abstract

Genome-wide association studies (GWAS) have become a standard approach for identifying quantitative trait loci associated with diverse phenotypic traits. Further investigation of the locus – specifically, the search for the causal gene and mutation – may present various challenges. One of the challenges is genetic heterogeneity (or locus heterogeneity), when alleles from different closely located genes can influence the same trait. Recently, using GWAS, we found the qDTF-7 locus on soybean chromosome 3, which is associated with flowering time under Novosibirsk conditions. Initially, we identified GmTOE1, an ortholog of TOE1 (TARGET OF EAT1), a known flowering-time regulator in Arabidopsis, as the most likely candidate gene for this locus. Four major haplotypes were identified in GmTOE1, which are associated with soybean flowering and maturity and are likely to provide soybean adaptation to northern latitudes. However, this gene showed only a very weak association with soybean flowering in the Novosibirsk region compared to the Oryol region, suggesting the presence of another gene within the locus that influences flowering time. We therefore re-analyzed genes in the qDTF-7 locus and identified GmRVE8c, an Arabidopsis RVE8 (REVEILLE 8) ortholog, located ~21 kb upstream of GmTOE1; RVE8 is a circadian clock component involved in plant development. After studying the natural variation of the GmRVE8c genes, we found four major haplotypes that arose due to three nonsynonymous substitutions and one 19-bp deletion leading to a frameshift. To identify three haplotypes, GmRVE8chap1, 3, 4, which are predominant in improved soybean cultivars, we developed DNA markers. Using these markers, we genotyped 129 soybean accessions, the developmental time of which had been studied in the Novosibirsk and Oryol regions. Using our data and data from SoyOmics, we found the GmRVE8chap3 and GmRVE8chap4 haplotypes to be associated with late flowering and maturity in soybean. The early-maturing haplotype GmRVE8chap1 is predominant in cultivars from northern regions and is likely associated with the adaptation of soybean to high latitudes. The GmRVE8chap4 haplotype is in complete linkage with the early-maturing allele GmTOE1C, whereas the GmRVE8chap3 haplotype shows strong linkage with the late maturing allele GmTOE1T. Furthermore, the ANOVA results indicate an interaction between GmRVE8c and E1, the major regulator of flowering in soybean. This interaction is manifested as a stronger effect of the GmRVE8chap3,4 haplotypes on flowering and maturity in the genetic background of the e1-as allele compared with E1. Together, these findings define a complex and intriguing locus, which may serve as a possible example of a genetically heterogeneous locus.

## Abstract (ru)

В настоящее время полногеномный анализ ассоциаций (GWAS) стал стандартным подходом для идентификации локусов количественных признаков, ассоциированных с различными фенотипическими признаками. При последующем изучении локуса, т. е. поиске гена и мутации, которые влияют на признак, можно столкнуться с разными проблемами. Одна из проблем – генетическая (или локусная) гетерогенность, ситуация, когда аллели из разных близко расположенных генов в одном локусе влияют на один признак. Ранее с помощью GWAS на хромосоме 3 сои мы обнаружили локус qDTF-7, ассоциированный с продолжительностью цветения в условиях Новосибирска. Первоначально для данного локуса в качестве наиболее вероятного гена-кандидата мы определили GmTOE1, ортолог TOE1 (TARGET OF EAT 1), известного регулятора цветения у арабидопсиса. У GmTOE1 были обнаружены четыре основных гаплотипа, которые ассоциированы с цветением и созреванием сои и, вероятно, связаны с адаптацией сои к северным широтам. Однако этот ген демонстрировал очень слабую ассоциацию с цветением сои в Новосибирской области по сравнению с Орловской, что указывало на наличие в составе локуса другого гена, который может влиять на цветение сои. Повторно проанализировав гены в составе локуса qDTF-7, мы примерно на 21 т. п. н. выше гена GmTOE1 обнаружили GmRVE8c, ортолог гена RVE8 (REVEILLE 8), который вовлечен в процессы развития у арабидопсиса в качестве компонента циркадных ритмов. Изучив естественное нуклеотидное разнообразие GmRVE8c, мы выявили четыре основных гаплотипа, которые возникли из-за трех однонуклеотидных замен и одной делеции длиной 19 п. н., приводящей к сдвигу рамки считывания. Для определения трех гаплотипов GmRVE8chap1, 3, 4, преобладающих в современных сортах сои, мы разработали ДНК-маркеры. С помощью этих маркеров мы генотипировали 129 сортообразцов сои, для которых ранее изучили продолжительность развития в условиях Новосибирской и Орловской областей. В результате с помощью наших сведений и данных из SoyOmics мы обнаружили ассоциацию гаплотипов GmRVE8chap3 и GmRVE8chap4 с поздним цветением и созреванием сои. Раннеспелый гаплотип GmRVE8chap1 преобладает в сортообразцах из северных регионов и, вероятно, связан с адаптацией сои к северным широтам. Гаплотип GmRVE8chap4 полностью сцеплен с раннеспелым аллелем GmTOE1С, а гаплотип GmRVE8chap3 сильно сцеплен с позднеспелым аллелем GmTOE1T. Кроме того, результат ANOVA показывает наличие взаимодействия GmRVE8c с главным регулятором цветения сои – геном E1. Данное взаимодействие выражается более сильным эффектом гаплотипов GmRVE8chap3, 4 на цветение и созревание на генетическом фоне аллеля e1-as в сравнении с E1. Все это формирует достаточно комплексный и интересный локус, который может выступать как возможный пример генетически гетерогенного локуса.

## Introduction

Advances in high-throughput plant phenotyping and genotyping methods, as well as the development of mathematical models that enable the identification of links between phenotype and genotype, have led to the discovery of numerous quantitative trait loci in plants (Zhang et al., 2022). The flowering and maturity time of soybean is no exception, and genes controlling these traits have been identified using GWAS (Genome-Wide Association Studies), for example: Tof5 (Dong et al., 2022), Tof8 (Li H. et al., 2023a), Tof13 (Li H. et al., 2023b), Tof16 (Dong et al., 2021), qFT13-3 (Li Y.F. et al., 2024), GmAP1d (Guo et al., 2024). Thus, the Tof16 locus was found to encode an ortholog of the Arabidopsis CCA1 (CIRCADIAN CLOCK ASSOCIATED 1) gene, which is a key component of the circadian clock and a transcription factor containing a DNA-binding MYB domain (Dong et al., 2021). Recessive tof16 alleles that partially or completely lose the original function of the encoded protein delay flowering and maturity time in soybean, which is more favorable for cultivars grown in southern latitudes (Dong et al., 2021).

A total of 54 CCA1-Like proteins have been identified in the soybean genome (Bian et al., 2017), four of which belong to the same phylogenetic clade and show the highest similarity to the Arabidopsis genes RVE4 (REVEILLE 4) and RVE8 (Bian et al., 2017; Shan et al., 2021; Bao et al., 2024). Loss of RVE8 function in Arabidopsis reduces flowering time under both short-day (SD) and long-day (LD) conditions, whereas overexpression delays flowering (Rawat et al., 2011). In addition to reduced flowering time, the rve4 6 8 and rve3 4 5 6 8 Arabidopsis mutants exhibit accelerated development and increased cell size due to disrupted expression of PIF4 (PHYTOCHROME INTERACTING FACTOR 4) and PIF5, as well as reduced proteasomes activity (Gray et al., 2017; Scandola et al., 2022). The functional role of RVE genes in flowering and maturity time in soybean is still poorly understood (Shan et al., 2021), although only recently it was shown that overexpression of GmMYB133 (the gene most closely related to Arabidopsis RVE5) and GmRVE8a in Arabidopsis leads to delayed flowering (Shan et al., 2021; Bao et al., 2024). Interestingly, overexpression of GmPIF4b in soybean accelerates not only flowering but also subsequent developmental stages (Arya et al., 2021). In soybean, a GmMYB133-PRR5-PIF4 module has also recently been proposed, as GmMYB133 in Arabidopsis can bind to the PRR5 (PSEUDO-RESPONSE REGULATOR 5) promoter, and PRR5 can, in turn, bind to PIF4 (Shan et al., 2021)

At present, genes and mutations affecting traits have been identified for only a relatively small number of quantitative trait loci in plants. Several challenges interfere with finemapping, that is, the identification of the gene and mutation within a locus that influence the expression of a trait (Clauw et al., 2024). One of the challenges is allelic heterogeneity, a situation in which different mutations in a single gene lead to the same or similar phenotype (Sasaki et al., 2021; Clauw et al., 2024; Liu H.-J. et al., 2024). One of the classic examples in plants is the multiple alleles of the FRI (FRIGIDA) gene in Arabidopsis, which have a confirmed effect on the expression of FLC (FLOWERING LOCUS C) but are not detected by GWAS unless these alleles are included as covariates in the statistical model (Atwell et al., 2010). Another less-studied challenge is genetic heterogeneity (or locus heterogeneity) – a situation in which alleles from different closely located genes at the same locus influence the same trait (Sasaki et al., 2021; Clauw et al., 2024; Liu H.-J. et al., 2024). Significantly fewer examples of genetic heterogeneity are currently known. Recently, E. Sasaki et al. (2021) characterized such a locus in Arabidopsis, showing that the association of the AOP2/ AOP3 (ALKENYL HYDROXALKYL PRODUCING) genes with flowering time can, in fact, be explained by two neighboring genes, NDX1 (NODULIN HOMEOBOX GENES 1) and GA1 (GIBBERELLIC ACID REQUIRING 1). A similar locus was also found in soybean, where pod shattering resistance was shown to arise from mutations in two nearby genes, Sh1 and Pdh1 (Li S. et al., 2024).

In this article, we discuss a possible example of a genetically heterogeneous locus in soybean. By reanalyzing genes within the soybean flowering-associated qDTF-7 locus, we identified a new candidate gene, GmRVE8c, located ~21 kb upstream of another strong candidate gene, GmTOE1. Mutations in GmRVE8c were found to form distinct haplotypes associated with soybean flowering and maturity. Furthermore, the effects of GmRVE8c haplotypes on flowering and maturity time depend on the allelic state of E1, the main regulator of flowering in soybean. Altogether, this forms a complex and intriguing locus, and our work may serve as a theoretical example of the natural mutations underlying associations identified through GWAS.

## Materials and methods

Plant materials and phenotypes. As plant material, we used 129 soybean accessions (63 cultivars and 66 breeding lines), which were described and used in our previous works (Perfil’ev et al., 2023, 2024). For these cultivars, the duration from emergence to flowering (DTF, days from emergence to flowering) and to maturity (DTM, days from emergence to maturity) was previously studied in Novosibirsk and Oryol in 2021 and 2022. The details of this field experiment were described in a previous work (Perfil’ev et al., 2023). We used BLUP (Best Linear Unbiased Prediction) values as phenotypes for these 129 accessions, which were calculated separately for each region, as described previously (Perfil’ev et al., 2024).

All available observations for BBD (Beginning bloom date) and MD (Pod maturity date) were downloaded from SoyOmics (https://ngdc.cncb.ac.cn/soyomics/index) (Liu Y. et al., 2023). BLUP values for these phenotypes were calculated using the “lmer” function from the lme4 package (Bates et al., 2015), with genotype, location, and year included as random effects in the model. The resulting BLUP values were used for further analyses.

Characterization of the qDTF-7 locus and natural variation in the GmRVE8c gene. The list of genes located within the qDTF-7 locus was obtained using SoyBase (https://www. soybase.org/).

Data on haplotypes of the GmRVE8c/SoyZH13_03G159500 gene within the coding sequence were obtained using the “Hap- Snap” module from SoyOmics. The settings in “HapSnap” were kept at default, except for the “Variation type” option, which excluded “Synonymous” and “Unclassed” variation.The ModiDB database was used to search for the main protein domains of GmRVE8c/Glyma.03G177300 (https:// mobidb.org/) (Piovesan et al., 2025).

Co-localization of qDTF-7 with previously identified QTLs was examined using the “BreedingTips” module from the SoyOmics website

DNA extraction, development of DNA markers, genotyping. Genomic DNA was extracted from 3–4-day-old seedlings grown in Petri dishes using a CTAB buffer (Rogers, Bendich, 1985).

To develop DNA markers for the GmRVE8c/ Glyma.03G177300 gene, the nucleotide sequence obtained from the Ensembl Plants database was used (https://plants. ensembl.org/index.html). UGENE v48.0 software was used for primer design (Okonechnikov et al., 2012) (http://ugene. net/ru/). The nucleotide sequences of the primers are provided in the Table.

**Table 1.**
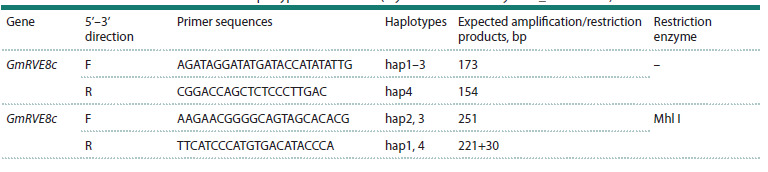
DNA markers used to determine the main haplotypes of GmRVE8c (Glyma.03G177300/SoyZH13_03G159500)

PCR was performed using BioMaster HS-Taq PCR-Color (2×) (BiolabMix, Novosibirsk, Russia). The mixture with a total volume of 25 μl contained 10 mM Tris-HCl, pH 8.5, 50 mM KCl, 0.1 % Tween 20, 2 mM MgCl2, 0.25 mM of each primer, 50–100 ng of genomic DNA, and 1 U Taq DNA polymerase. The primers used are presented in the Table. PCR conditions: 95 °C, 5 min; (95 °C, 15 s; 55 °C, 10 s; 72 °C, 20 s) for 34 cycles; 72 °C, 1 min.

Digestion of PCR products using the restriction enzyme Mhl I (SibEnzyme, Novosibirsk, Russia) was carried out in a reaction mixture with a total volume of 20 μL, which contained: 8 μl of PCR product; 2 μl 10x buffer recommended by the manufacturer; 1 U enzyme; 10 μl ddH20. Restriction and amplification products were separated on a 2–2.5 % agarose gel with ethidium bromide. Electrophoresis results were photographed using Gel Doc™ XR+ (BioRad, USA). The “Step100+50” DNA ladder (BiolabMix, Novosibirsk, Russia) was used as a molecular size marker

Genotypes of the E1 and GmTOE1 genes for 129 soybean accessions were previously obtained and published (Perfil’ev et al., 2023, 2024). For improved cultivars from the SoyOmics database, genotypes for the mutations soy8699425 for the E1/SoyZH13_06G195900 gene and soy4989324 for the GmTOE1/SoyZH13_03G159700 gene were obtained using the “HapSnap” module, and for the mutation soy8699425, they were additionally recoded as C → e1-as, G → E1 allele

Statistical analysis. The association of GmRVE8c haplotypes with flowering and maturity time was analyzed using the basic function “aov” in R version 4.4.2 (https://www.r-project. org/). Multiple haplotype comparisons were performed using the “TukeyHSD” function. Boxplots and heatmaps illustrating the results of multiple comparisons were generated using the R packages “ggplot2” (Wickham, 2016) and “ggcorplot” (Kassambara, 2023).

## Results

**Revisiting the qDTF-7 locus identified a new candidate gene, GmRVE8c**

Previously, using the FarmCPU multilocus model on soybean chromosome 3, we identified a QTN (Gm03_40959110_A_G) that was associated with flowering time in Novosibirsk conditions (Perfil’ev et al., 2024). This QTN was located within a 553 kb LD block, which was designated as the qDTF-7 locus (QTL days from emergence to flowering 7) (Perfil’ev et al., 2024). This locus contains 71 genes (Table S1 in Supplementary Materials)1, and we initially identified Glyma.03G177500 (hereafter referred to as GmTOE1) as the most likely candidate gene (Perfil’ev et al., 2024). However, this gene exhibited a much weaker association with soybean flowering in Novosibirsk compared to Oryol (Perfil’ev et al., 2024). Therefore, we decided to revisit the genes within this locus more closely and, as a result, found Glyma.03G177300. This gene encodes one of four soybean homologs that are most closely related to the Arabidopsis genes RVE8 (REVEILLE 8) and RVE4 (Bao et al., 2024). In this article, the Glyma.03G177300 gene will be referred to as GmRVE8c.

Supplementary Materials are available in the online version of the paper: https://vavilovj-icg.ru/download/pict-2026-30/appx3.zip


In Arabidopsis, the RVE8 gene is a known component of the circadian clock and also functions to delay flowering (Rawat et al., 2011). Overexpression of one of the soybean RVE8 orthologs (GmRVE8a) in Arabidopsis leads to delayed flowering (Bao et al., 2024). Collectively, these findings suggest that GmRVE8c is a candidate gene for regulating flowering and maturity in soybean.

**Association of GmRVE8c haplotypes with flowering and maturity of soybean**

Using the SoyOmics database, we studied natural variation in the coding region of the GmRVE8c gene. As a result, we found four haplotypes, which arose due to one deletion causing a frameshift and three nonsynonymous substitutions (Table S2). This deletion is predicted to cause a frameshift after four amino acids, likely leading to a complete loss of the gene’s original function. All nonsynonymous substitutions found are located outside the predicted MYB DNA-binding domains of the protein (Fig. S1). It is difficult to assume whether these substitutions have consequences for the structure and function of the encoded protein

**Fig. 1.**
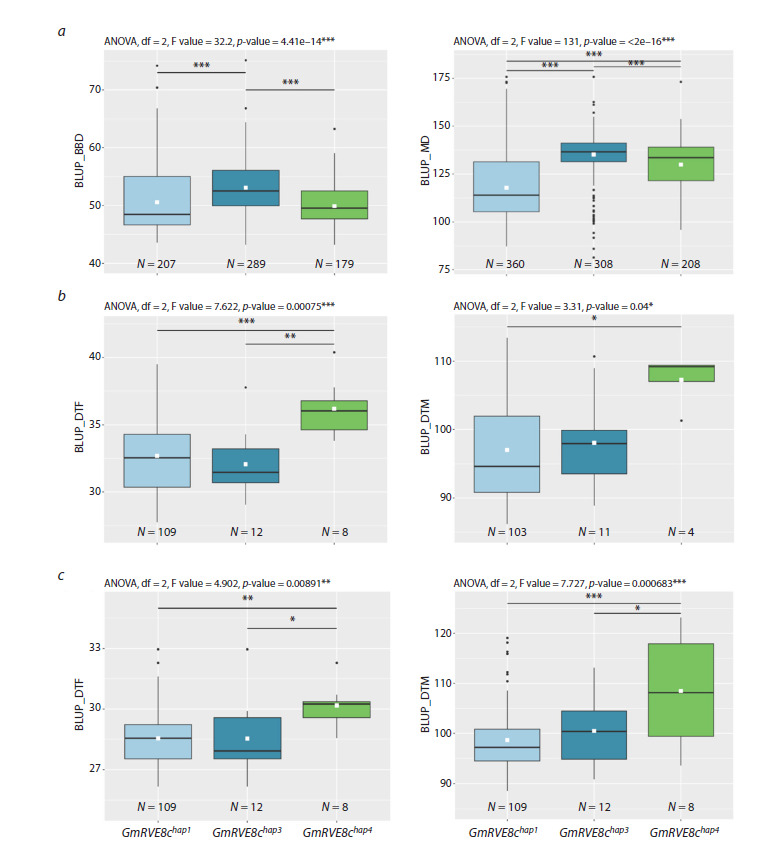
Association of haplotypes GmRVE8c with flowering and maturity time. a – association of haplotypes GmRVE8c with BLUP_BBD and BLUP_MD; b – association of haplotypes GmRVE8c with BLUP_DTF and BLUP_DTM in Novosibirsk; c – association of haplotypes GmRVE8c with BLUP_DTF and BLUP_DTM in Oryol. White squares on the boxplots indicate the mean value. Asterisks indicate significant differences between the compared genotypes: *** p <0.001; ** p < 0.01; * p < 0.05.

We studied the association of three common haplotypes with BLUP_BBD and BLUP_MD in improved soybean cultivars. In cultivated varieties, according to the results of the association analysis for BLUP_BBD, cultivars carrying GmRVE8chap3flower significantly later than those carrying GmRVE8chap1 and GmRVE8chap4 (Fig. 1a). According to the results of the association analysis for BLUP_MD, cultivars carrying GmRVE8chap3 and GmRVE8chap4 mature significantly later than those carrying GmRVE8chap1 (Fig. 1a).

To identify these three haplotypes, we developed DNA markers for the soy32494699 and soy4988787 mutations (see the Table, Fig. S2) and used them to genotype 129 soybean accessions from our collection, in which the qDTF-7 locus was initially identified. As a result, GmRVE8chap1 was found in 109 accessions, while the remaining 12 and 8 carried haplotypes GmRVE8chap3 and GmRVE8chap4, respectively (Table S3). In addition, GmRVE8c shows a significant association with BLUP_DTF and BLUP_MD under the conditions of the Novosibirsk and Oryol regions, and accessions carrying the GmRVE8chap4 haplotype flower and mature significantly later (Fig. 1b, c).

In cultivars Graciya and VNIIS-1, we previously identified the GmTOE1T allele (Perfil’ev et al., 2024); however, genotyping for GmRVE8c revealed the GmRVE8chap4 haplotype in these cultivars, which was unexpected, since GmRVE8chap4 is linked to the GmTOE1С allele. It should be noted that the GmRVE8c gene is physically located very close to GmTOE1, making the occurrence of crossover genotypes GmRVE8chap4 GmTOE1T extremely unlikely. For example, no such genotype was found in the SoyOmics database. Therefore, we re-extracted DNA from these cultivars and genotyped them using a previously developed DNA marker for the GmTOE1C allele (Perfil’ev et al., 2024), confirming the presence of the GmTOE1C allele in these cultivars (Table S3).

**The GmRVE8c gene exhibits an epistatic interaction with the E1 gene and is also located near another flowering regulator, GmTOE1**

In soybean, GmCCA1s (CIRCADIAN CLOCK ASSOCIATED 1) genes promote flowering by repressing the expression of the major flowering repressor, E1 (Dong et al., 2021). RVE genes belong to a family of transcription factors with CCA1-like MYB domains, so we suggested that the function of GmRVE8c in flowering regulation might also depend on the E1 gene.

The distance between GmRVE8c and GmTOE1 is approximately 21 kb, and GmTOE1 is nearly the immediate downstream gene following GmRVE8c in the 5ʹ–3ʹ direction (Table S1). GmTOE1 harbors natural variation that is associated with flowering and maturity time in soybeans (Perfil’ev et al., 2024). Previously, we identified four main haplotypes, including two early-maturing and two late-maturing haplotypes (hereafter referred to as the GmTOE1С and GmTOE1T alleles, respectively), which differ from each other by the nucleotide substitution T316C, resulting in the replacement of serine with proline at the 106th amino acid (Perfil’ev et al., 2024).

We examined the association of GmRVE8c haplotypes, taking into account the E1 and GmTOE1 genetic back-grounds, using data from SoyOmics and from our experiment (Fig. S3–S6). The ANOVA results show a significant interaction between GmRVE8c and E1, based on data from SoyOmics (Tables S4, S5). This indirectly suggests the presence of an epistatic interaction between GmRVE8c and E1. Analysis of the established associations using data from SoyOmics showed that, on the e1-as allele background, the GmRVE8chap3 and GmRVE8chap4 haplotypes have a stronger effect on flowering and maturity time (Fig. S3a, b). In our collection, plants carrying the GmRVE8chap3 and GmRVE8chap4 haplotypes also carry the e1-as or e1-nl alleles, and plants with GmRVE8chap4 exhibit later flowering and maturity compared to other genotypes (Fig. S3c, d).

According to BLUP_BBD data, plants with GmRVE8chap1 GmTOE1T and GmRVE8chap3 GmTOE1T flower significantly later than those with GmRVE8chap4 GmTOE1C and GmRVE8chap1 GmTOE1C (Fig. S4a). A similar pattern is observed for BLUP_MD,with the exception of the GmRVE8chap4 GmTOE1C genotype, which matures later than GmRVE8chap1 GmTOE1C (Fig. S4a).According to data from our collection, plants with GmRVE8chap4GmTOE1С flower and mature significantly later than those with GmRVE8chap1 GmTOE1С in both regions (Fig. S4b–c). According to BLUP_MD and BLUP_BBD, plants with the GmRVE8chap1 GmTOE1C genotype flower and mature significantly earlier than those with GmRVE8chap1 GmTOE1T (Fig. S4a). According to the data from our collection, plants with the GmRVE8chap1 Gm- TOE1C genotype flower significantly earlier than those with GmRVE8chap1 GmTOE1T, but only under the conditions of Oryol (Fig. S4b). The GmTOE1C allele is associated with early flowering and maturity, as we described previously, and GmRVE8chap4 appears to partially restore the early-maturing phenotype of GmTOE1C plants, making them late-maturing (Fig. S4a).

When considering all three genes simultaneously, we interpreted the results by comparing genotypes that differ from each other at only one gene. According to BLUP_BBD and BLUP_MD, plants with the GmRVE8chap1 e1-as GmTOE1С genotype flower and mature later than those with GmRVE8chap4 e1-as GmTOE1С (Fig. S5). According to BLUP_BBD, there are no significant differences between genotypes GmRVE8chap1E1 GmTOE1С and GmRVE8chap1 E1 GmTOE1T, or between GmRVE8chap1 e1-as GmTOE1С and GmRVE8chap1 e1-as GmTOE1T (Fig. S5a). According to BLUP_MD, of the two genotypes, only GmRVE8chap1 E1 GmTOE1С and GmRVE8chap1 E1 GmTOE1T differ significantly from each other (Fig. S5b). In our collection, plants with the GmRVE8chap1 e1-nl GmTOE1С genotype flower significantly earlier than those with GmRVE8chap1 e1-nl GmTOE1T under Oryol conditions (Fig. S6c). In addition, genotypes with GmRVE8chap4 e1-as GmTOE1С flower and mature later than the other genotypes (Fig. S6). Overall (although not completely) this repeats the result of the two-gene analysis from the previous paragraph.

There are also significant differences in BLUP_MD and BLUP_BBD between genotypes GmRVE8chap1 E1 GmTOE1С and GmRVE8chap4 E1 GmTOE1С, with plants carrying GmRVE8chap4 maturing later but flowering slightly earlier (Fig. S5). In the background of the e1-as allele, the GmRVE8chap4 haplotype shows a stronger effect on BLUP_ BBD (Fig. S5a). For the GmRVE8chap3 haplotype, a similar pattern is observed, with no significant differences in BLUP_BBD and BLUP_MD between the GmRVE8chap3 E1 GmTOE1T and GmRVE8chap1 E1 GmTOE1T genotypes (Fig. S5). However, a significant difference in BLUP_MD is observed between the GmRVE8chap3 e1-as GmTOE1T and GmRVE8chap1 e1-as GmTOE1T genotypes (Fig. S5b). In our collection, plants carrying the GmRVE8chap4 haplotype, even in the presence of the null e1-nl allele, show late flowering and maturity (Fig. S5).

**Distribution of GmRVE8c haplotypes in soybean accessions of different origin**

**Fig. 2.**
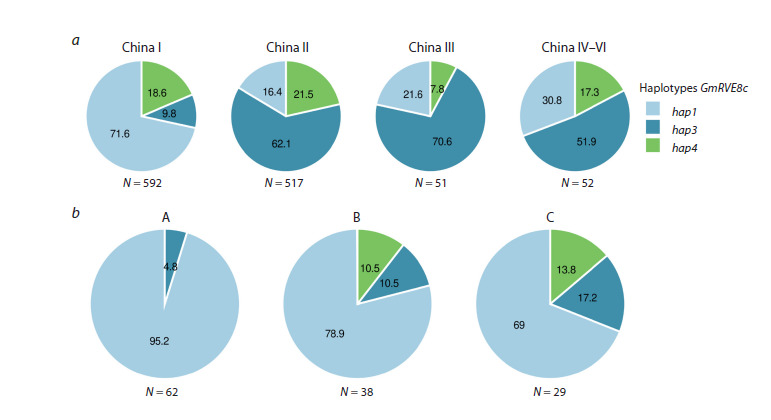
Distribution of GmRVE8c haplotypes: a – in improved soybean cultivars from China (China I designates northern provinces of China, and China II, III, IV–VI designate southern provinces); b – distribution of GmRVE8c haplotypes in three groups of soybean accessions: A – from West Siberia (Novosibirsk and Omsk regions); B – from other Russian regions; and C – from other countries. Numbers on pie charts indicate percentages; N denotes the number of accessions.

For the GmRVE8c gene, we examined haplotype frequencies in soybean cultivars from China across three major soybean growing regions (China I, China II, and China III) and one mixed region (China IV–VI), as well as in our collection, which was divided into three main groups: A – from West Siberia (Novosibirsk and Omsk regions); B – from other Russian regions; and C – from other countries (Fig. 2).

The GmRVE8chap1 haplotype predominates in cultivars from northern China, whereas GmRVE8chap3 predominates in the other China regions. In our collection, accessions from Western Siberia predominantly carry the GmRVE8chap1 haplotype, whereas the late-maturing GmRVE8chap4 haplotype is present only in accessions from other regions of Russia and from other countries (Fig. 2b). This demonstrates the role of GmRVE8c haplotypes in the adaptation of soybean to different cultivation regions.

## Discussion

**The GmRVE8c gene is a new candidate gene for regulating flowering and maturity in soybeans**

We identified the Glyma.03G177300/GmRVE8c gene as a new candidate gene for the previously found qDTF-7 locus associated with soybean flowering (Perfil’ev et al., 2024). GmRVE8c was found to have four major haplotypes resulting from three single nucleotide substitutions and a 19-bp deletion (Table S2). Among the mutations identified in GmRVE8c, the soy32494699 substitution (a 19-bp deletion) in the GmRVE8chap4 haplotype may lead to a complete loss of the encoded protein’s original function due to a frameshift occurring after the fourth amino acid (Table S2). The remaining three GmRVE8chap1–3 haplotypes arose due to three nonsynonymous substitutions (Table S2). Although all three substitutions are located outside the predicted DNA-binding domains (Fig. S1), they may still affect the 3D structure of the protein or its interactions with other proteins (Fig. S1). Notably, two of the identified mutations are located within the predicted LIP (Linear Interacting Peptides) regions of the protein (Fig. S1). A complete loss of GmRVE8c function appears to result in delayed soybean development, as plants carrying the GmRVE8chap4 haplotype exhibit late flowering and maturity (Fig. 1). It is also possible that the GmRVE8chap3 haplotype has also lost the original function of the protein, since, according to SoyOmics data, samples carrying this haplotype also exhibit late flowering and maturity (Fig. 1a).

The frequency of GmRVE8c haplotypes shifts from wild to cultivated soybean; compared with wild soybean, the Gm- RVE8chap2 haplotype is almost absent in landrace and modern cultivars (Table S2). Moreover, the GmRVE8chap4 haplotype is not detected in the wild soybean population and likely arose and was selected after soybean domestication (Table S2). The distribution of GmRVE8c haplotypes across different cultivation regions shows that the early-maturing haplotype GmRVE8chap1 predominates in cultivars from northern China, whereas GmRVE8chap3 predominates in other regions (Fig. 2a); similarly, in our collection, almost all cultivars from Western Siberia carry the GmRVE8chap1 haplotype (Fig. 2b). This highlights the role of GmRVE8c haplotypes in the adaptation of soybean to different cultivation regions. Due to its association with late flowering and maturity, the GmRVE8chap4 haplotype is unfavorable for northern latitudes but, conversely, advantageous for southern latitudes. We believe that GmRVE8chap4 could be valuable in breeding soybean cultivars adapted to southern regions

**The GmRVE8c gene shows epistatic interaction with the E1 gene**

The RVE gene family encodes transcription factors with CCA1-like MYB domains and is closely related to the CCA1 (CIRCADIAN CLOCK ASSOCIATED 1) and LHY (LATE ELONGATED HYPOCOTYL) genes (Rawat et al., 2009). In soybean, the CCA1/LHY genes are known regulators of flowering, and under SD conditions, GmLHYs directly bind to the E1 promoter and repress its expression, thereby accelerating flowering, whereas loss of GmLHYs function delays flowering (Dong et al., 2021). Based on this, we hypothesized that the role of GmRVE8c in flowering regulation may also depend on the E1 gene. ANOVA results based on SoyOmics data show a significant interaction between E1 and GmRVE8c, suggesting that GmRVE8c may also act as a repressor of the E1 gene (Tables S3, S4). Association analysis of these data shows that the GmRVE8chap3 and GmRVE8chap4 haplotypes more strongly delay flowering and maturity in the background of the semifunctional e1-as allele (Fig. S4a, b). This is an unexpected situation, because if we assume that GmRVE8c can suppress the expression of the E1 gene, then in the presence of the weakly functional e1-as allele, loss of GmRVE8c function should not lead to a phenotypic change, or at least the effect should be weaker. Similarly, the effect on the flowering time of the loci/genes Tof16/LHYa, Tof8/FKF1b, and J/ELF3 under the background of the e1-as allele completely disappeared or was reduced (Lu S. et al., 2017; Dong et al., 2021; Li H. et al., 2023a). Phenotypic analysis in our collection shows that the GmRVE8chap4 haplotype affects flowering and maturity even in the presence of the e1-nl allele, which has a deletion of the entire E1 gene (Fig. S3c, d). It is likely that, under our conditions, GmRVE8c may be involved in the transcriptional regulation of the entire E1 gene family (E1, E1La, E1Lb), or that GmRVE8c controls flowering time through an E1- independent mechanism.

In Arabidopsis, proteins LNK1 (NIGHT LIGHT–INDUCIBLE AND CLOCK-REGULATED 1) and LNK2 recruit RVE4 and RVE8 to activate transcription of TOC1 (TIMING OF CAB EXPRESSION 1) and PRR5 (PSEUDO-RESPONSE REGULATOR 5), and loss of function of LNK1–4 results in delayed flowering under LD conditions (Xie et al., 2014; De Leone et al., 2018). Knockout of the four GmLNK2s orthologs in soybean accelerates flowering under LD conditions due to reduced expression of the E1 gene (Li Z. et al., 2021). A characteristic feature of LNK genes is the absence of their own DNA-binding domains, for which they recruit CCA1, LHY, RVE4 and RVE8, and switch them from repressors to activators of transcription (Xie et al., 2014). Further studies will clarify whether the function of GmRVE8c in flowering control depends on the E1 gene family and whether GmLNK2s proteins recruit GmRVE8s proteins to regulate E1 gene transcription.

**The qDTF-7 locus as a possible example of a heterogeneous locus in soybean**

Locus or genetic heterogeneity is a situation in which alleles from different closely located genes at the same locus influence the same trait. For the qDTF-7 locus, we previously proposed a strong candidate gene, GmTOE1 (Perfil’ev et al., 2024), which has been shown to play a role in the regulation of flowering in soybean (Wang T. et al., 2016). In this study, we identified a new candidate gene, GmRVE8c, located ~21 kb upstream of GmTOE1, which may also be involved in the regulation of flowering and maturity in soybean. Using the data available to us, we attempted to analyze how mutations in the GmRVE8c and GmTOE1 genes correspond to each other. In the genetic background of the early-flowering/maturing GmTOE1C allele, the late-maturing GmRVE8chap4 haplotype arose. We did not find any GmRVE8chap4 GmTOE1T genotypes in either the SoyOmics database or our collection (Fig. S4). Based on SoyOmics phenotypic data, the effect of the GmRVE8chap4 haplotype appears to partially restore the effect of the earlymaturing GmTOE1C allele on flowering and maturity time (Fig. S4a, S5a, b).

A similar pattern is observed in our collection; however, the GmRVE8chap4 haplotype shows a much stronger effect on flowering and maturity time than either GmTOE1C or GmTOE1T (Fig. S4b, c, S6a, b). GmTOE1 alleles have a minor effect on flowering and maturity under our conditions; for example, genotype GmRVE8chap1 GmTOE1С differs significantly from GmRVE8chap1 GmTOE1С only for BLUP_ DTF in Oryol conditions (Fig. S4b, c, S6a, b). Interestingly, the GmRVE8chap3 haplotype predominates in the background of the GmTOE1T allele, and, for example, in the SoyOmics database and in our collection, we found only two accessions with the GmRVE8chap3 GmTOE1С genotype (Fig. S4). Therefore, it is difficult to determine whether the delayed flowering and maturity are caused by GmRVE8chap3 or by GmTOE1T, although SoyOmics data suggest that both genes influence these traits (Fig. S4a, S5). However, phenotypic analysis in our collection did not reveal any significant effect of GmRVE8chap3 on flowering and maturity time (Fig. S4b, c, S6a, b).

It should be mentioned that the mutations detected in the GmRVE8c and GmTOE1 genes require functional validation to confirm whether they actually affect the phenotype. If this proves true, the qDTF-7 locus could serve as an interesting example of how natural mutations shape phenotypic diversity in plants, particularly in soybean.

**The influence of the GmRVE8c gene on other soybean traits**

The early-maturing GmTOE1C allele likely contributes to soybean adaptation to northern latitudes by reducing flowering time (Perfil’ev et al., 2024). In contrast, the GmRVE8chap4 haplotype is unfavorable for northern latitudes due to its association with late flowering and maturity, whereas in our collection, all accessions from Western Siberia carry the early-maturing GmRVE8chap1 haplotype (Fig. 2). As discussed above, GmRVE8chap4 appears to restore the early-maturing phenotype of GmTOE1C, raising the question of why the GmRVE8chap4 mutation arose in the presence of the early-maturing GmTOE1C allele. A possible explanation for this is the pleiotropic effect of GmRVE8c on other soybean traits. Interestingly, several loci associated with soybean drought tolerance have been identified on chromosome 3 using bi-parental QTL mapping (Table S6). The genes GmRVE8c and GmTOE1 are also positioned within these loci. However, it should be noted that these loci also contain the known drought tolerance regulator GmSALT3 (Shi et al., 2018; Guan et al., 2021).

Overexpression of GmRVE8a in Arabidopsis enhances drought and salt tolerance, and GmRVE8a can interact with the GmNAC019 protein (Bao et al., 2024), which is involved in the ABA-mediated drought response (Hoang et al., 2019). GmLHYs genes are also involved in the ABA-mediated drought response in soybean, and knockout of all four GmLHYs genes enhances drought tolerance (Wang K. et al., 2021). Recently, it has been shown that the soybean circadian clock component GmPRR3b directly suppresses the expression of GmABF3 (ABSCISIC ACID-RESPONSIVE ELEMENT-BINDING FACTOR 3), the overexpression of which enhances drought tolerance in soybean (Li C. et al., 2024). We hypothesize that loss of GmRVE8c function may also enhance drought tolerance in soybean. Interestingly, in Arabidopsis, the TOE1 and TOE2 genes have been shown to play key roles in drought escape, and loss of TOE1/2 function accelerates flowering but reduces drought tolerance (Chen et al., 2025). For the early-maturing GmTOE1C allele, we assume at least a partial loss of function of the encoded protein (Perfil’ev et al., 2024). It is possible that the GmRVE8chap4 haplotype arose to compensate for drought sensitivity in plants carrying the GmTOE1C allele. In our view, this is an intriguing hypothesis that warrants further exploration.

According to Table S6, the GmRVE8c gene colocalizes with loci associated with plant height and weight, oil and isoflavone content, seed thickness, and resistance to soybean cyst nematode. Theoretically, GmRVE8c may also influence some of these traits, such as plant height, and weight, potentially through regulation of the PRR5–PIF4 module, as has been proposed for GmMYB133 (Shan et al., 2021). GmMYB133 has also been shown to be a positive regulator of isoflavone biosynthesis in soybean (Bian et al., 2018). In Arabidopsis, rve 4 6 8 mutants exhibit altered metabolism of carbohydrates, fatty acids, and lipids, as well as reduced proteasome activity, which leads to an increase in cell size (Scandola et al., 2022). Various circadian clock genes have been studied and shown to contribute to plant defense against pathogens (Lu H. et al., 2017). In particular, the role of GmCCA1-1 in soybean response to infection by the nematode Heterodera glycines has recently been demonstrated (Niraula et al., 2022). All of this makes GmRVE8c an interesting gene for further study, not only in the context of soybean flowering and maturity but also for other agriculturally important traits.

## Conclusion

In this study, we identified a new candidate gene, GmRVE8c, and two of its haplotypes, GmRVE8chap3 and GmRVE8chap4, which are associated with late flowering and maturity in soybean. In addition, to distinguish these haplotypes from each other, we developed DNA markers that can be used for markerassisted selection in soybean. The GmRVE8c gene is located in close proximity to another soybean flowering regulator, GmTOE1, which also has mutations associated with flowering and maturity time. GmTOE1 and GmRVE8c are likely to form a genetically heterogeneous locus, i. e. a locus in which alleles of different nearby genes influence the same trait. Also, the influence of the GmRVE8chap3 and GmRVE8chap4 haplotypes on the flowering and maturity time shows partial dependence on the allelic state of the E1 gene. Altogether, these findings make qDTF-7 a highly complex and intriguing locus, and we plan to further validate the mutations in GmRVE8c and GmTOE1 using hybrid soybean populations

## Conflict of interest

The authors declare no conflict of interest.

## References

Arya H., Singh M.B., Bhalla P.L. Overexpression of PIF4 affects plant
morphology and accelerates reproductive phase transitions in soybean.
Food Energy Secur. 2021;10(3):e291. doi 10.1002/fes3.291

Atwell S., Huang Y.S., Vilhjálmsson B.J., Willems G., Horton M., Li Y.,
Meng D., … Weigel D., Marjoram P., Borevitz J.O., Bergelson J.,
Nordborg M. Genome-wide association study of 107 phenotypes in
Arabidopsis thaliana inbred lines. Nature. 2010;465(7298):627-631.
doi 10.1038/nature08800

Bao G., Sun G., Wang J., Shi T., Xu X., Zhai L., Bian S., Li X. Soybean
RVE8a confers salt and drought tolerance in Arabidopsis. Biochem
Biophys Res Communs. 2024;704:149660. doi 10.1016/j.bbrc.
2024.149660

Bates D., Mächler M., Bolker B., Walker S. Fitting linear mixed-effects
models using lme4. J Stat Software. 2015;67(1):1-48. doi 10.18637/
jss.v067.i01

Bian S., Jin D., Li R., Xie X., Gao G., Sun W., Li Y., Zhai L., Li X.
Genome-wide analysis of CCA1-like proteins in soybean and functional
characterization of GmMYB138a. Int J Mol Sci. 2017;18(10):
2040. doi 10.3390/ijms18102040

Bian S., Li R., Xia S., Liu Y., Jin D., Xie X., Dhaubhadel S., Zhai L.,
Wang J., Li X. Soybean CCA1-like MYB transcription factor
GmMYB133
modulates isoflavonoid biosynthesis. Biochem Biophys
Res Commun. 2018;507(1-4):324-329. doi 10.1016/j.bbrc.2018.
11.033

Chen W., Wang T., Li X., Feng J., Liu Q., Xu Z., You Q., Yang L.,
Liu L., Chen S., Yue Z., Wang H., Yu D. Arabidopsis RGLG1/2
regulate flowering time under different soil moisture conditions by
affecting the protein stability of TOE1/2. New Phytol. 2025;246(4):
1609-1626. doi 10.1111/nph.70073

Clauw P., Ellis T.J., Liu H.-J., Sasaki E. Beyond the standard GWAS –
a guide for plant biologists. Plant Cell Physiol. 2025;66(4):431-443.
doi 10.1093/pcp/pcae079

De Leone M.J., Hernando C.E., Romanowski A., García-Hourquet M.,
Careno D., Casal J., Rugnone M., Mora-García S., Yanovsky M.J.
The LNK gene family: at the crossroad between light signaling and
the circadian clock. Genes. 2019;10(1):2. doi 10.3390/genes10010002

Dong L., Fang C., Cheng Q., Su T., Kou K., Kong L., Zhang C., …
Yuan X., Weller J.L., Lu S., Kong F., Liu B. Genetic basis and adaptation
trajectory of soybean from its temperate origin to tropics. Nat
Commun. 2021;12:5445. doi 10.1038/s41467-021-25800-3

Dong L., Cheng Q., Fang C., Kong L., Yang H., Hou Z., Li Y., …
Tang Y., Zhao X., Lu S., Liu B., Kong F. Parallel selection of distinct
Tof5 alleles drove the adaptation of cultivated and wild soybean to
high latitudes. Mol Plant. 2022;15(2):308-321. doi 10.1016/j.molp.
2021.10.004

Gray J.A., Shalit-Kaneh A., Chu D.N., Hsu P.Y., Harmer S.L. The
REVEILLE clock genes inhibit growth of juvenile and adult plants
by control of cell size. Plant Physiol. 2017;173(4):2308-2322. doi
10.1104/pp.17.00109

Guan R., Yu L., Liu X., Li M., Chang R., Gilliham M., Qiu L. Selection
of the salt tolerance gene GmSALT3 during six decades of soybean
breeding in China. Front Plant Sci. 2021;12:794241. doi 10.3389/
fpls.2021.794241

Guo S., Li Y., Qiu H., Hu G., Zhao C., Wang R., Zhang H., Tian Y.,
Li X., Liu B., Li Ying-hui, Qiu L. GmAP1d regulates flowering time
under long-day photoperiods in soybean. Crop J. 2024;12(3):845-
855. doi 10.1016/j.cj.2024.03.004

Hoang X., Nguyen N., Nguyen Y.-N., Watanabe Y., Tran L.-S. The soybean
GmNAC019 transcription factor mediates drought tolerance in
Arabidopsis in an abscisic acid-dependent manner. Int J Mol Sci.
2019;21(1):286. doi 10.3390/ijms21010286

Kassambara A. ggcorrplot: Visualization of a Correlation Matrix Using
“ggplot2”. 2023. R package version 0.1.4.999. Available: https://
github.com/kassambara/ggcorrplot

Li C., Chen Y., Hu Q., Yang X., Zhao Y., Lin Y., Yuan J., Gu J., Li Y.,
He J., Wang D., Liu B., Wang Z.-Y. PSEUDO-RESPONSE REGULATOR
3b and transcription factor ABF3 modulate abscisic
acid-dependent drought stress response in soybean. Plant Physiol.
2024;195(4):3053-3071. doi 10.1093/plphys/kiae269

Li H., Du H., He M., Wang J., Wang F., Yuan W., Huang Z., … Liu B.,
Kong F., Fang C., Zhao X., Yu D. Natural variation of FKF1 controls
flowering and adaptation during soybean domestication and
improvement. New Phytol. 2023a;238(4):1671-1684. doi 10.1111/
nph.18826

Li H., Du H., Huang Z., He M., Kong L., Fang C., Chen L., Yang H.,
Zhang Y., Liu B., Kong F., Zhao X. The AP2/ERF transcription factor
TOE4b regulates photoperiodic flowering and grain yield per
plant in soybean. Plant Biotechnol J. 2023b;21(8):1682-1694. doi
10.1111/pbi.14069

Li H., Du H., Huang Z., He M., Kong L., Fang C., Chen L., Yang H.,
Zhang Y., Liu B., Kong F., Zhao X. The AP2/ERF transcription factor
TOE4b regulates photoperiodic flowering and grain yield per
plant in soybean. Plant Biotechnol J. 2023b;21(8):1682-1694. doi
10.1111/pbi.14069

Li S., Wang W., Sun L., Zhu H., Hou R., Zhang H., Tang X., Clark C.B.,
Swarm S.A., Nelson R.L., Ma J. Artificial selection of mutations in
two nearby genes gave rise to shattering resistance in soybean. Nat
Commun. 2024;15:7588. doi 10.1038/s41467-024-52044-8

Li Y.F., Zhang L., Wang J., Wang X., Guo S., Xu Z.J., Li D., Liu Z.,
Li Y.H., Liu B., Qiu L.J. Flowering time regulator qFT13‐3 involved
in soybean adaptation to high latitudes. Plant Biotechnol J. 2024;
22(5):1164-1176. doi 10.1111/pbi.14254

Li Z., Cheng Q., Gan Z., Hou Z., Zhang Y., Li Y., Li H., … Kou K.,
Wang L., Kong F., Liu B., Dong L. Multiplex CRISPR/Cas9-mediated
knockout of soybean LNK2 advances flowering time. Crop J.
2021;9(4):767-776. doi 10.1016/j.cj.2020.09.005

Liu H.-J., Swarts K., Xu S., Yan J., Nordborg M. On the contribution of
genetic heterogeneity to complex traits. bioRxiv. 2024. doi 10.1101/
2024.03.27.586967

Liu Y., Zhang Y., Liu X., Shen Y., Tian D., Yang X., Liu S., Ni L.,
Zhang Z., Song S., Tian Z. SoyOmics: a deeply integrated database
on soybean multi-omics. Mol Plant. 2023;16(5):794-797. doi
10.1016/j.molp.2023.03.011

Lu H., McClung C.R., Zhang C. Tick Tock: circadian regulation of
plant innate immunity. Annu Rev Phytopathol. 2017;55:287-311. doi
10.1146/annurev-phyto-080516-035451

Lu S., Zhao X., Hu Y., Liu S., Nan H., Li X., Fang C., … Weller J.L.,
Liu B., Hou X., Tian Z., Kong F. Natural variation at the soybean
J locus improves adaptation to the tropics and enhances yield. Nat
Genet. 2017;49(5):773-779. doi 10.1038/ng.3819

Niraula P.M., McNeece B.T., Sharma K., Alkharouf N.W., Lawrence
K.S., Klink V.P. The central circadian regulator CCA1 functions
in Glycine max during defense to a root pathogen, regulating
the expression of genes acting in effector triggered immunity (ETI)
and cell wall metabolism. Plant Physiol Biochem. 2022;185:198-
220. doi 10.1016/j.plaphy.2022.05.028

Okonechnikov K., Golosova O., Fursov M., UGENE team. Unipro
UGENE: a unified bioinformatics toolkit. Bioinformatics. 2012;
28(8):1166-1167. doi 10.1093/bioinformatics/bts091

Perfil’ev R., Shcherban A., Potapov D., Maksimenko K., Kiryukhin S.,
Gurinovich S., Panarina V., Polyudina R., Salina E. Impact of allelic
variation in maturity genes E1–E4 on soybean adaptation to Central and West Siberian regions of Russia. Agriculture. 2023;13(6):1251.
doi 10.3390/agriculture13061251

Perfil’ev R., Shcherban A., Potapov D., Maksimenko K., Kiryukhin S.,
Gurinovich S., Panarina V., Polyudina R., Salina E. Genome-wide
association study revealed some new candidate genes associated
with flowering and maturity time of soybean in Central and West
Siberian regions of Russia. Front Plant Sci. 2024;15:1463121. doi
10.3389/fpls.2024.1463121

Piovesan D., Del Conte A., Mehdiabadi M., Aspromonte M.C.,
Blum M., Tesei G., von Bülow S., Lindorff-Larsen K., Tosatto S.C.E.
MOBIDB in 2025: integrating ensemble properties and function annotations
for intrinsically disordered proteins. Nucleic Acids Res.
2025;53(D1):D495-D503. doi 10.1093/nar/gkae969

Rawat R., Schwartz J., Jones M.A., Sairanen I., Cheng Y., Andersson
C.R., Zhao Y., Ljung K., Harmer S.L. REVEILLE1, a Myb-like
transcription factor, integrates the circadian clock and auxin pathways.
Proc Natl Acad Sci USA. 2009;106(39):16883-16888. doi
10.1073/pnas.0813035106

Rawat R., Takahashi N., Hsu P.Y., Jones M.A., Schwartz J., Salemi
M.R., Phinney B.S., Harmer S.L. REVEILLE8 and PSEUDOREPONSE
REGULATOR5 form a negative feedback loop within
the Arabidopsis circadian clock. PLoS Genet. 2011;7(3):e1001350.
doi 10.1371/journal.pgen.1001350

Rogers S.O., Bendich A.J. Extraction of DNA from milligram amounts
of fresh, herbarium and mummified plant tissues. Plant Mol Biol.
1985;5(2):69-76. doi 10.1007/BF00020088

Sasaki E., Köcher T., Filiault D.L., Nordborg M. Revisiting a GWAS
peak in Arabidopsis thaliana reveals possible confounding by genetic
heterogeneity. Heredity. 2021;127(3):245-252. doi 10.1038/
s41437-021-00456-3

Scandola S., Mehta D., Li Q., Rodriguez Gallo M.C., Castillo B.,
Uhrig
R.G. Multi-omic analysis shows REVEILLE clock genes are
involved in carbohydrate metabolism and proteasome function.
Plant Physiol. 2022;190(2):1005-1023. doi 10.1093/plphys/kiac269

Shan B., Wang W., Cao J., Xia S., Li R., Bian S., Li X. Soybean
GmMYB133
inhibits hypocotyl elongation and confers salt tolerance
in Arabidopsis. Front Plant Sci. 2021;12:764074. doi 10.3389/
fpls.2021.764074

Shi X., Yan L., Yang C., Yan W., Moseley D.O., Wang T., Liu B., Di R.,
Chen P., Zhang M. Identification of a major quantitative trait locus
underlying salt tolerance in ‘Jidou 12’ soybean cultivar. BMC Res
Notes. 2018;11:95. doi 10.1186/s13104-018-3202-3

Wang K., Bu T., Cheng Q., Dong L., Su T., Chen Z., Kong F., Gong Z.,
Liu B., Li M. Two homologous LHY pairs negatively control soybean
drought tolerance by repressing the abscisic acid responses.
New Phytol. 2021;229(5):2660-2675. doi 10.1111/nph.17019

Wang T., Sun M.-Y., Wang X.-S., Li W.-B., Li Y.-G. Over-expression
of GmGIa-regulated soybean miR172a confers early flowering in
transgenic Arabidopsis thaliana. Int J Mol Sci. 2016;17(5):645. doi
10.3390/ijms17050645

Wickham H. ggplot2: Elegant Graphics for Data Analysis. Springer,
2016. doi 10.1007/978-3-319-24277-4

Xie Q., Wang P., Liu X., Yuan L., Wang L., Zhang C., Li Y., Xing H.,
Zhi L., Yue Z., Zhao C., McClung C.R., Xu X. LNK1 and LNK2 are
transcriptional coactivators in the Arabidopsis circadian oscillator.
Plant Cell. 2014;26(7):2843-2857. doi 10.1105/tpc.114.126573

Zhang M., Liu S., Wang Z., Yuan Y., Zhang Z., Liang Q., Yang X.,
Duan Z., Liu Y., Kong F., Liu B., Ren B., Tian Z. Progress in soybean
functional genomics over the past decade. Plant Biotechnol J.
2022;20(2):256-282. doi 10.1111/pbi.13682

